# Dynamics of reproductive genetic technologies: Perspectives of professional stakeholders

**DOI:** 10.1371/journal.pone.0269719

**Published:** 2022-06-21

**Authors:** Ivy van Dijke, Carla G. van El, Phillis Lakeman, Mariëtte Goddijn, Tessel Rigter, Martina C. Cornel, Lidewij Henneman

**Affiliations:** 1 Department of Human Genetics and Amsterdam Reproduction & Development Research Institute, Amsterdam UMC, Vrije Universiteit Amsterdam, Amsterdam, The Netherlands; 2 Amsterdam UMC, University of Amsterdam, Center for Reproductive Medicine and Amsterdam Reproduction & Development Research Institute, Amsterdam, The Netherlands; 3 Department of Human Genetics and Amsterdam Public Health Research Institute, Amsterdam UMC, Vrije Universiteit Amsterdam, Amsterdam, The Netherlands; 4 Department of Human Genetics and Amsterdam Reproduction & Development Research Institute, Amsterdam UMC, University of Amsterdam, Amsterdam, The Netherlands; Flinders University, AUSTRALIA

## Abstract

Reproductive and genetic medicine are evolving rapidly, and new technologies are already impacting current practices. This includes technologies that can identify a couples’ risk of having a child with a genetic disorder. Responsible implementation of new technologies requires evaluation of safety and ethics. Valuable insights for shaping governance processes are provided by various stakeholders involved, including healthcare professionals. Their willingness to adopt these technologies and guide the necessary systemic changes is required for the successful implementation of these technologies. In this study, twenty-one semi-structured interviews were conducted with professionals from different disciplines in the field of reproductive and genetic healthcare in the Netherlands. Three emerging technologies were discussed: expanded carrier screening (ECS), non-invasive prenatal diagnosis (NIPD) and germline genome editing (GGE). By probing stakeholders’ views, we explored how culture, structure and practice in healthcare is being shaped by innovations and changing dynamics in genetic and reproductive medicine. The general consensus was that the implementation of reproductive genetic technologies nationwide is a slow process in Dutch healthcare. A “typical Dutch approach” emerged that is characterized by restrictive legislation, broad support for people living with disabilities, values of an egalitarian society and limited commercialisation. Different scenarios for embedding ECS in future practice were envisioned, while implementation of NIPD in clinical practice was considered obvious. Views on GGE varied among stakeholders. Previous implementation examples in the Netherlands suggest introduction of new technology involves an organized collective learning process, with pilot studies and stepwise implementation. In addition, introducing and scaling up new technologies is complex due to perceived barriers from the legislative framework and the complex relationship between the government and stakeholders in this area. This paper describes how the international trends and advances of technologies are expected to manifest itself in a national setting.

## Introduction

The development and introduction of new technologies in healthcare is constantly evolving, including reproductive and genetic medicine. Premature implementation of new technologies can be an actual risk for (future) recipients in reproductive medicine [1], therefore thorough safety and ethical evaluation of technologies is needed [2]. Implementing new technologies in an existing field involves changes and transitions for a broad range of stakeholders in organizing (structure), thinking (culture) and doing (practice) [3]. All stakeholders involved should thus be engaged to ensure responsible implementation. Two leading professional organisations, the European Society of Human Genetics (ESHG) and European Society for Human Reproduction and Embryology (ESHRE), published a statement in 2018 on emerging topics at the interface of reproductive and genetic medicine [4]. Among these, several new technologies were listed (Box 1), including expanded carrier screening for assessing reproductive genetic risk, non-invasive prenatal testing and diagnosis, and germline genome editing. There are calls for regulation of these, and other, new reproductive genetic technologies [2,4–6], supported by arguments that these impact not only end-users but future generations [7]. Technological advances surpassing capabilities of current clinical practices cause shifts in among others, information provision, uptake, availability, and costs. Therefore, it is relevant to study the interplay of these technologies in more detail to anticipate possible shifts in use, which are not straightforward due to various stages of development and/or implementation within the healthcare system, and consequential impacts on uptake.

**Box 1. Definitions of technologies discussed in the interviews**

**Table:** 

**Expanded carrier screening (ECS**) determines the carrier status of an individual / couple for multiple recessive disorders simultaneously, and aims to identify couples at increased risk for having affected offspring in order to inform reproductive decision-making. ECS is ideally performed before pregnancy. Couples from the general population can opt for ECS, regardless of their risk based on ancestry. **Germline Genome Editing (GGE)** involves modification of the DNA of early embryos, sperm or oocytes, potentially preventing genetic disorders. Genetic changes can be passed down to future generations. Currently, GGE is not allowed and it is not expected to be available in the near future. **Non-Invasive Prenatal Diagnosis (NIPD)** screens for specific autosomal recessive or dominant disorders by analysing fetal cell free DNA (cfDNA) isolated from maternal plasma. Pregnant women with an increased risk of having an affected child in the absence or presence of a positive family history or after ultrasound with abnormal results suggesting dominant disorders are eligible. NIPD can be a non-invasive alternative for PND (see below). It is expected that NIPD will be available for a growing number of disorders. **Non-Invasive Prenatal Testing (NIPT)** allows for the screening of fetal aneuploidies (including trisomy 13,18 and 21) by analysing cfDNA isolated from maternal plasma of pregnant women. Detection of aneuploidy allows women to prepare for the birth of an affected child or to terminate the pregnancy. NIPT is available in many countries. **Prenatal Diagnosis (PND)** refers to invasive procedures like chorionic villus sampling and amniocentesis that are preformed prenatally to detect specific genetic and/or chromosomal abnormalities in high risk pregnancies. The test result allows women to prepare for the birth of an affected child or to terminate the pregnancy. PND is available in many countries. **Preimplantation Genetic Testing (PGT)** involves genetic analysis of a single cell or small number of cells of an early stage embryo for a specific known genetic or chromosomal disorder. The embryos are created through in vitro fertilization with intracytoplasmic sperm injection (IVF/ICSI). An unaffected embryo is transferred to the uterus. Confirmation of the PGT results by PND is offered because of a small residual risk of a PGT misdiagnosis. PGT is available in many countries for couples at increased risk.

Preconception carrier screening informs prospective parents if they are a carrier couple for a specific autosomal recessive or X-linked disorder and thus obtain information about their increased risk of having a child with that disorder before pregnancy. Traditionally, preconception carrier screening was offered for a limited number of disorders to certain high-risk groups based on ancestry. Next-generation genome sequencing has made it possible to expand the number of conditions screened, hence the term expanded carrier screening (ECS). Evolving sequencing technology also has lowered costs to the point that ECS can be, in principle, offered to all prospective parents [8]. One option for couples who have an increased risk for having an affected child is preimplantation genetic testing (PGT), in which unaffected embryos are selected prior to implantation in the womb. During pregnancy, another option is invasive prenatal diagnosis (PND), where the chorionic villus or amniotic fluid is sampled to test for a known familial disorder in the foetus. If more carrier couples are identified with ECS, the need for PGT, PND or non-invasive prenatal diagnosis (NIPD) could increase [9]. NIPD, which is based on analysis of foetal cell-free DNA (cfDNA) from maternal blood, is a non-invasive method that will likely soon become widely available and for many conditions, opening the door to analysis of the entire foetal genome [10]. CfDNA sequencing also allows screening for specific foetal chromosomal aneuploidies in non-invasive prenatal testing (NIPT), a technology which is already broadly implemented internationally [11,12]. Both NIPT and NIPD are expanding to include more disorders, as the sequencing costs continue to decrease. Another emerging technology that was discussed by the ESHG and ESHRE [4] is germline genome editing (GGE). With GGE, the DNA of embryos or germ cells could be modified to prevent genetic disease. Great commotion was caused across the globe when the first case (and last up to now) of genome edited babies in China was reported [13]. However, a global moratorium is in place that prohibits GGE in a clinical setting (i.e. transfer of embryo to the uterus) as current technological practices may be harmful to the embryo and raise serious ethical and fundamental concerns [14].

The successful implementation of new technologies is largely dependent on adoption by crucial stakeholders [15]. In order to guide responsible introduction of new technologies, governance is necessary. Assessing stakeholders’ views is critical to obtain insight into the deployment process and assess necessary changes, moreover, to raise awareness among stakeholders and wider audiences. Table 1 presents an introduction to the key characteristics of the Dutch healthcare system, and an overview of the currently available reproductive genetic technologies, along with emerging technologies possibly available in the future.

**Table 1:** The context of reproductive genetic technologies in the Netherlands, discussed in this study.

Healthcare system in the Netherlands	Current application of reproductive genetic technologies	
The healthcare system in the Netherlands is controlled by the government together with private health insurance companies: a public-private system. The government has the responsibility of monitoring the quality of care and setting healthcare priorities. Everyone in the Netherlands is obliged to join a health insurance company for a basic package of care (expanded at will, at people’s own expense) to access healthcare. The Health Insurance Act (Zorgverzekeringswet) is in place since 2006. In this basic healthcare package, a consultation with the general practitioner, prescription drugs or hospital visits, including a clinical geneticist consultation, are included [16]. It should be noted that every person of 18 years or older pays at least a yearly out-of-pocket amount of €385 before being reimbursed for healthcare. General practitioner visits are exempt.	ECS*	Since 2020, a professional guideline for carrier screening in the Netherlands has been developed to indicate which tests are needed for which at-risk groups (i.e. ancestry-based, consanguinity) [17]. As of today, carrier screening is not broadly offered to the general population, i.e. people without an increased risk, and private commercial providers are forbidden to offer this due to legislation [18]. Since 2016, two (out of the country’s eight) University hospitals [19,20] offer an ECS panel of 50–70 conditions available to the general population. At the time of this study, these tests are not reimbursed by national healthcare, and costs range between €650-€1100.
	**GGE**	GGE is currently illegal in the Netherlands, similar to many other countries. Research on human embryos is not allowed beyond 14 days and embryos cannot be created for research purposes. Initiated by the Ministry of Health Welfare and Sports and other organisations, ‘The Dutch DNA-dialogue project’ about GGE took place in 2019–2020. The dialogue was organised among different stakeholder groups including the general public, prospective parents, and children at primary and high school level in order to collect a broad range of views from the Dutch general public on GGE and invite stakeholders to form opinions on the matter [21].
	**NIPD**	At the time of this study, NIPD for the detection of monogenic disorders is not yet available in the Netherlands. A pilot study to evaluate the diagnostic accuracy of NIPD for monogenic disorders around 8–10 weeks of gestation is ongoing, but suffers from low participation and was temporarily halted due to the COVID-19 pandemic. It is expected that NIPD will eventually be available to couples with a known increased risk on having an affected child.
	**NIPT**	Implementation of NIPT for all pregnant women in the national prenatal screening program has been executed in a study (TRIDENT-2 [22]). Professionals have organized a Dutch NIPT Consortium that works closely with the government [11]. This allows for thorough evaluation of the implementation process, including ongoing research into the cultural acceptability of NIPT, and the counselling and training of healthcare professionals. At the time of this study, the NIPT is available to all women as a first trimester screening test (cost €175).
	**PGT**	PGT has been available in the Netherlands for over 25 years. An independent committee is reviewing the eligibility of new indications. There are four academic hospitals that offer counselling for PGT treatment. Only one centre (Maastricht UMC) has a government permit to perform the actual diagnostic DNA testing on the embryos. Three cycles of in vitro fertilization along with intracytoplasmic sperm injection are reimbursed.
	**PND**	To identify a (specific) chromosomal abnormality, a familial pathogenic DNA variant, or a genetic syndrome, invasive prenatal diagnostic testing (chorionic villus sampling and amniocentesis) is offered to high-risk couples and reimbursed.

Abbreviations: ECS = expanded carrier screening, GGE = germline genome editing, NIPT = non-invasive prenatal testing, NIPD = non-invasive prenatal diagnosis, PGT = preimplantation genetic testing and PND = prenatal diagnosis.

The emerging technologies discussed in this study are expected to have a large impact on the field of reproductive medicine and genetics in the near future [4]. This makes exploration of Dutch stakeholders’ perspectives and expectations concerning the future development and possible implementation of ECS, NIPD and GGE in the existing reproductive genetic healthcare field extremely relevant. We discuss how international trends and new technologies are expected to manifest in a national setting.

## Materials and methods

### Study design

A qualitative study design using semi-structured interviews was applied for this study. The Medical Ethical Committee of the Amsterdam University Medical Center (location AMC) approved the study protocol and exempted this study from ethical review (W18_054).

### Theoretical framework

**Fig 1:**
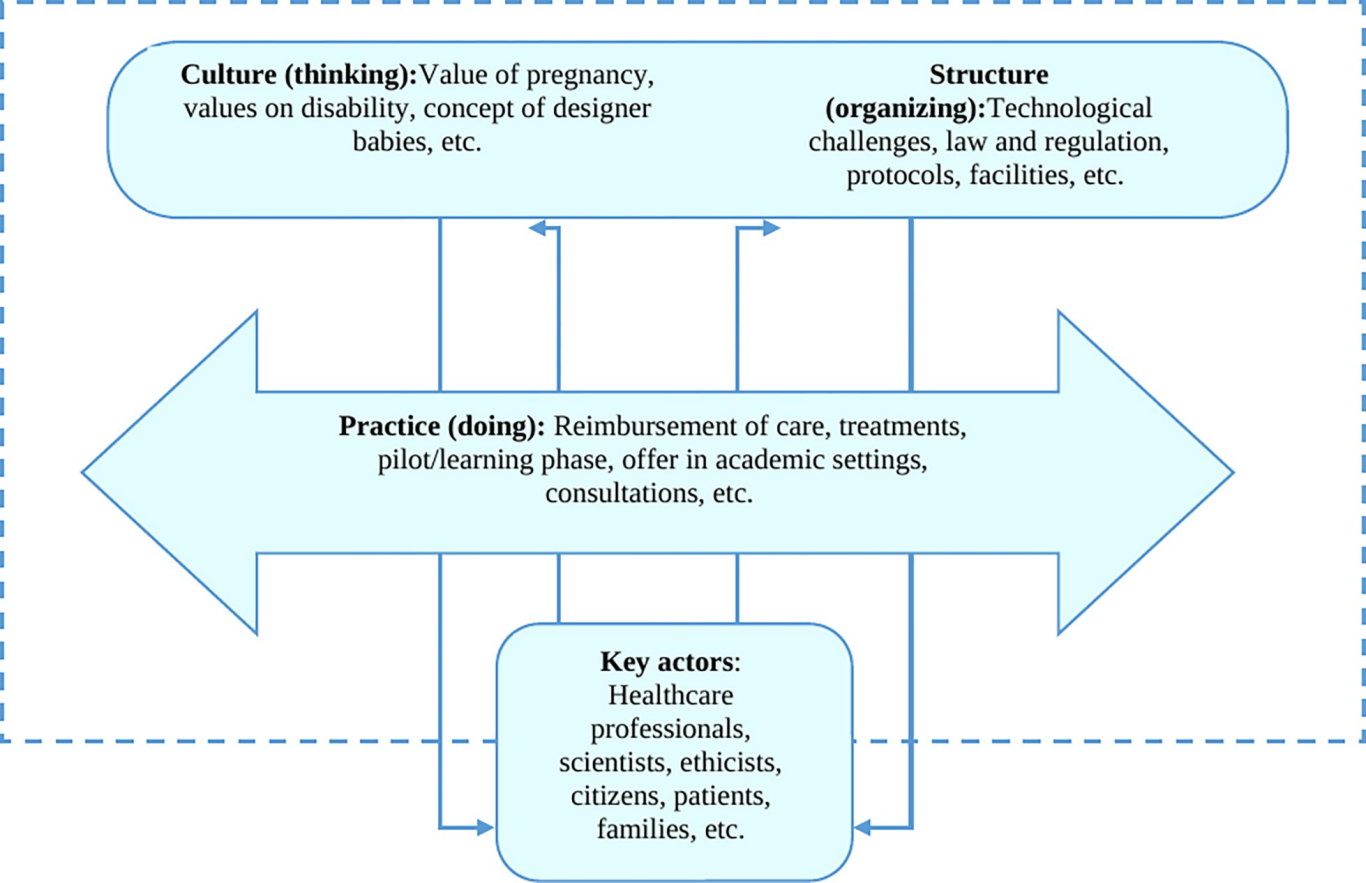
The existing system of reproductive medicine: Operationalization of the constellation concept into structure, culture and practice, adapted from Rigter [25] and van Raak [23].

Two theoretical models, the Constellation Perspective and the Network of Actors model, were used for the study design and the interpretation of findings. The Constellation Perspective [23] argues that a (healthcare) system can be seen as a constellation of interrelated practices and relevant structuring elements, and can be described by its (i) structure (organizational and power structures), (ii) culture (values and thinking) and (iii) practice (actions and implementation) (Fig 1). According to this model, actors (stakeholders) determine structure and culture. Actors are known to generally be hesitant to change, for example to adopt a new technology that is (not yet) concordant with the structure or culture of the existing system. When new technologies are developed and implemented in an existing (healthcare) system, transition occurs when fundamental changes in all three aspects of the system happen. Key actors in the field can either hinder or facilitate change, and act as change agents [3,24].

**Fig 2:**
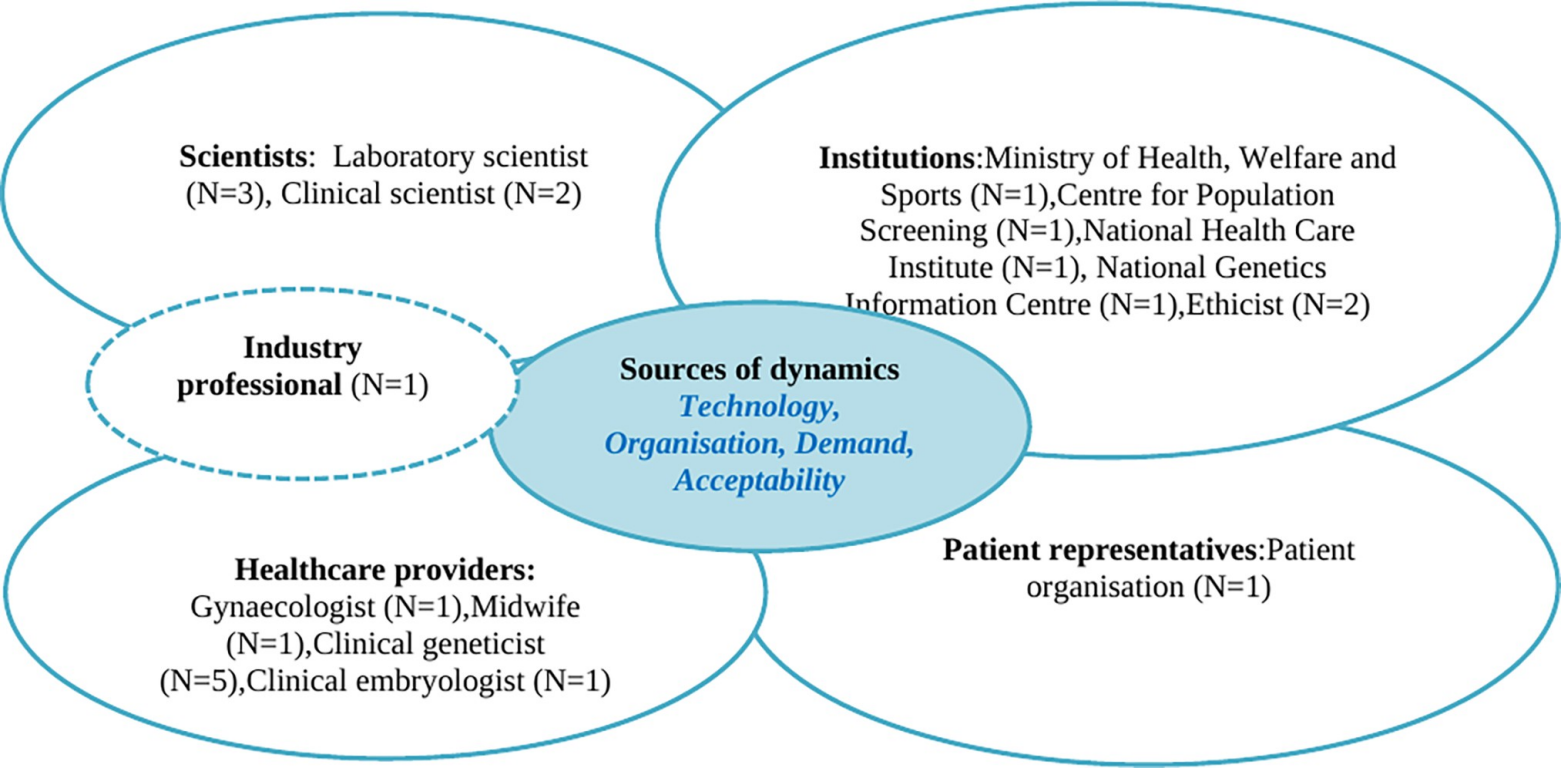
Network of Actors model, adapted from Achterbergh et al. [26]. The numbers of interviewees per stakeholder group are indicated with N = x.

The Network of Actors model can be used to identify and describe the key actors involved in the process of development and implementation of new technologies [26]. Moreover, this model is helpful to gain in depth understanding of technology development and implementation by focusing on the roles of stakeholders and their interactions. According to this model the different actors are divided in four categories: (i) the scientists who develop and do research into the *technology*, (ii) policymakers and ethicists who decide whether a technology is *acceptable* and should be made available, (iii) the healthcare providers who *organise* implementation of a technology in the actual healthcare system and offer it to recipients, and, last but not least, (iv) the citizens and patients who may use or *demand* a technology (Fig 2). The existing model does not include the industry; however, we believe this stakeholder group is of importance when discussing new technologies, both for development and implementation in health care and therefore we have invited someone from that field. The Network of Actors model facilitates exploring expectations and actions of the various stakeholders when attuning the use of technologies in a changing field. It should be noted that, in practice, stakeholders may have different roles to fulfil at the same time, e.g. working as a healthcare provider and policy adviser to the government. The sources of dynamics and roles/categories of actors involved are interrelated and merged in Fig 2 [3,26].

### Participants and procedure

A total number of 28 key stakeholders were identified with purposive sampling strategy using the Network of Actors model and 21 stakeholders were interviewed. Representing healthcare professionals included gynaecologists (n = 1), midwives (n = 1), clinical geneticists (n = 5) and clinical embryologists (n = 1), researchers included laboratory (n = 3) and clinical (n = 2) scientists; professionals from industry (n = 1), institutions included governmental (n = 2) and health insurance institutions (n = 1), ethicists (n = 2), legal experts (n = 1), and representatives from patient organisations (n = 1) (Fig 2). To achieve a spread of participants throughout the country, stakeholders working in different (academic) hospitals and institutions were invited. It should be noted that participants had different expertise and knowledge concerning the discussed technologies (i.e. some participants work solely in the field of PGT). The perspectives of couples who face an increased risk of having affected offspring, representing the stakeholders that may voice “demand” for these techniques, have been published elsewhere [27]. For this study, patient perspectives were therefore represented by contacting an alliance organisation for patients with rare and genetic diseases. Relevant stakeholders were contacted through email with an invitation letter, four stakeholders, among which three were healthcare professionals and one government employee replied that they did not consider themselves suitable for participation in this interview study, and three stakeholders, working for institutions and in healthcare, did not reply. From December 2019 until February 2021, twenty-one interviews of approximately 45 minutes were conducted (n = 5 face-to face and n = 16 online due to the COVID-19 pandemic) by one researcher (I.D.) who is trained in conducting interviews. Respondents gave verbal consent to participate which was recorded. All respondents were based in the Netherlands and/or affiliated with Dutch institutions.

### Interview guide

The following topics were discussed in the interviews: (i) Stakeholders’ own role, their current activities, their responsibilities and observed demand for these developments, (ii) expectations regarding the impact of the technologies on the existing field and current practice, and (iii) expectations regarding the dynamics between current and future technologies (i.e. ECS, NIPD/NIPT, PND, PGT, GGE).

### Data analysis

All interviews were recorded, using a recording device, after participants’ verbal consent and transcribed verbatim. For collecting and analysing qualitative data, we adhered to the COREQ checklist [28], supplemental material. The qualitative data software Atlas.ti 8 was used for analysis. First, open coding was performed and the perceived relevant items of the data were marked in the transcripts. Second, thematic content analysis was conducted. To increase validity, five transcripts were coded by two researchers independently [C.E. and I.D.]. The codes were compared, discussed and adapted until agreement was reached. All other transcripts were coded by one researcher [I.D.]. Themes and topics were selected for several reasons: most expressive, repeatedly identified, or remarkable cases. The quotes that were considered most relevant for illustration of the results were translated into English. When relevant, we tried to indicate which group particularly hold certain views. The stakeholder group and the interview number (participant; P1, 2, 3…) are indicated with the quotes.

## Results

The results of this study are structured into two identified themes. The first theme, “The typical Dutch approach”, illustrates the views on the current (reproductive genetic) healthcare system in the Netherlands by describing experiences of stakeholders with earlier implementation of genetic technologies in terms of current *practice*, and important *structural* and *cultural* elements. The second theme, “Moving forward with new technologies”, discusses the expectations concerning ECS, NIPD and GGE and their potential impact in detail.

### Theme 1: The typical Dutch approach

Participants framed the dynamics of new technologies within a cultural background. The typical Dutch approach emerged as a theme from discussing previous examples and experiences and looking forward on the new technologies discussing ECS, NIPD and GGE. The Dutch approach can be characterised as including careful consideration and restrictive legislation. Important values of the Dutch approach are the importance of an egalitarian society, autonomy and support for people with disabilities. It was mentioned that the Netherlands acts carefully, but as a consequence is not a pioneer in reproductive technology worldwide. They argued that technologies tend to be implemented only under certain conditions imposed by the government. Legislation, such as the Dutch Population Screening Act, contributes to accurate implementation of screening and aims to protect people against potential harm [29]. Several respondents called this a “conservative approach” when it comes to research and implementation of new (reproductive) technologies:

*“We have a government that handles the changes it wishes to pursue very responsibly*. *In the Netherlands*, *change is never immediate and even the smallest decisions get weighed and measured for pros and cons*. *(…) Look*, *we don’t usually lead the way*: *we like rules*, *laws and discussion too much for that*, *but we are never really behind the curve*. *But we’re not forerunners*.*”* P9, institution.

This sometimes frustrates stakeholders as they believe that certain legislation is too strict, hindering research and innovation and risking progress within the country:

*“I don’t understand why some processes here are so slow*, *it frustrates me*. *I contact a colleague abroad and hear that some techniques or tests are already being offered on a structural basis*. *We [referring to the Dutch government] are sometimes too strict*.*”* P4, healthcare provider.

In terms of actors influencing the reproductive healthcare system, some interviewees believed that professionals working in the field, especially healthcare providers, have an important role in the organization and implementation of new technologies, with the support of government policy. Stakeholders explain professional groups and consortia working in the field see it as their responsibility to develop guidelines based on state-of-the-art knowledge, in order to ensure people receive the best available care.

Participants believed that the political landscape has some influence as well. After elections, the Dutch government changes every four years and, according to some participants, this heavily influences the healthcare system. For example, if more conservative parties are elected, the availability or progress of implementing certain reproductive technologies may be affected. One respondent argued that once benefits of a certain technology are proven, it should be available to people, regardless of the political nature of the government:

*“It seems to me that a government which is unable to call upon Christian or other conservative beliefs would have a hard time stopping such change(s)*. *So*, *I assume that all these developments*, *when safe and effective*, *would in some way manage to find a spot in the healthcare system*.*”* P13, healthcare provider

Some stakeholders were somewhat hesitant describing their own role in the implementation of new technologies. However, all believed that participating in research was an important societal responsibility when working in the field of reproductive and genetic care.

### Cultural background

Implementation of new genetic technologies are greatly influenced by the cultural background, as was mentioned by participants. Several norms and values were mentioned that are characteristic of Dutch culture: (i) An egalitarian society, (ii) Concern about medicalisation of pregnancy and autonomous decisions, and (iii) Support for people living with disabilities.

**(i) The importance of an egalitarian society**. It was considered important by stakeholders to maintain healthcare services that are accessible for all. Some described this as an “Egalitarian society”:

*“In the Netherlands*, *we have a pretty egalitarian society*, *causing people to quickly call out*: *‘Hey*, *but this shouldn’t just be for the happy few*?*”* P14, institution.

An example listed by some of the participants was that the Netherlands was one of the first countries to integrate NIPT into routine prenatal care, making it widely available for all pregnant women, albeit with a fee that may deter a proportion of women (Table 1). Another example mentioned by healthcare providers was the three IVF/ICSI cycles within the PGT process that are reimbursed for most couples who face no medical, personal (e.g. age, obesity) and/or social contraindications. A member from an institution mentioned that this egalitarian approach sometimes creates difficulties for the government, for example, in a possible scenario where ECS is offered to everyone and fully reimbursed by the government:

“*On what grounds do you decide that a technique should be collectively offered*?*”* P12, institution.

**(ii) Concern about medicalisation of pregnancy and autonomous decisions.** Stakeholders brought up the societal discussions and concerns about **(**over)medicalization, this involves situations where more medical care is applied to a health condition than is required or recommended to achieve better health. Earlier discussions about the medicalisation of pregnancy have been central to, for example, a cautious implementation of prenatal screening in the Netherlands. Some stakeholders argued couples have ‘the right not to know’ and should be able to opt out of screening. An important related concept in reproductive decision making is autonomy. Stakeholders agreed that everyone should be able to make their own autonomous decisions, and in the Netherlands, this includes declining tests or information about tests. Current policy and counselling guidelines in the Netherlands are organized to support this. For instance, in prenatal screening, women are asked if they want to receive information about screening rather than being offered the screening directly. Moreover, feelings of any pressure to opt for certain technologies should be avoided, a member of an institution stated:

*“I do think that it is very important that people receive proper support in this selection process [of opting for a specific technology] and that they do not experience any pressure*. *It is important to pay attention to the benefits as well as the potential harms*.*”* P16, institution.

**(iii) Support for people living with disabilities.** All stakeholders agreed that the support for people living with disabilities is well established in the Dutch culture and healthcare. Some stakeholders thought this support could be threatened by the implementation of new technologies. When one can avoid having an affected child, treatments would still need to remain accessible.

*“I really believe in prevention*. *But if people do not want that*, *the facilities should remain available to take care of people with certain conditions*. *We have to ensure the continued existence of services*.” P4, healthcare provider.

Noticeably, respect for people with disabilities was often mentioned as another reason why the Dutch government is hesitant and careful in implementing preventative technologies like preconception carrier screening for the general public. In contrast, some others thought the attitude towards disabilities is sometimes ‘romanticized’ by favourable portrayal in the media, and this may lead to the general public underestimating the seriousness of some conditions.

### Theme 2: Moving forward with new technologies

The expectations of stakeholders concerning ECS, NIPD and GGE are structured according to the three elements of the Constellation Perspective model; *practice*, *structure* and *culture*. In general, stakeholders mentioned that attention should be paid to the costs of emerging technologies. In the preconception context, stakeholders pointed to the lack of genetic literacy in the general public and the need to reach and inform couples that are not pregnant yet about for example the availability of ECS. The growing number of available reproductive technologies was said to possibly hinder autonomous decision making, highlighting the need to provide counselling to support the process.

### Expectations of expanded carrier screening (ECS)

#### Practice: Several possible future scenarios

The professional stakeholders interviewed outlined several possible scenarios for implementing ECS for the general population. Some mentioned that ECS can be embedded within fertility clinics, where prospective parents could be informed about the availability of the ECS test:

*“I think it [ECS] should rather be integrated in reproductive medicine than in general healthcare*, *since prospective parents visiting fertility clinics have a clear child wish*.*”* P19, institution.

Another suggested scenario was to incorporate ECS in general practice. It could be offered actively by a general practitioner, by inviting all patients of reproductive age to participate, or passively through a website or flyers.

#### Structure: Expected changes

Several respondents believed that ECS will become an important new technology in reproductive medicine in the upcoming years. In terms of structure, some stakeholders thought that ECS should be integrated with other aspects of preconception care, like lifestyle advice and assessment of medical risk factors:

“*ECS needs to be as established—just like taking folic acid when planning a pregnancy*.*”* P18, healthcare provider.

Healthcare providers warned that the current capacity of PND and PGT would not be sufficient if ECS becomes mainstream. A stakeholder suggested that the role of geneticists could be expanded to provide training and support to other healthcare providers, in addition to counselling patients, which would require cultural and structural changes in the workplace. If ECS is implemented nationally, the costs of the test were frequently mentioned as a potential barrier, as not all couples may be able to afford it if not reimbursed:

*“A shift towards preconceptional care will majorly depend on the accompanying costs of such a change*. *I think we should realise that as long as ECS is not covered by insurance*, *and people would have to pay for it themselves*, *then only a select group of our society can use it*.*”* P5, scientist.

According to the stakeholders, ideally ECS should be fully reimbursed, however, this would be a major expense for the healthcare budget of the government. In addition to the expanded testing, healthcare providers will need additional education to provide counselling for ECS, one participant said. It was also noted that the test itself will likely become cheaper over time and therefore more accessible.

#### Culture: Needs for cultural changes

Culturally, preconception care, or visiting a healthcare professional when planning a pregnancy, is not yet embedded in Dutch society, stakeholders mentioned. It was suggested, this could be related to the generally negative attitude towards medicalisation of pregnancy. It was stressed that genetic literacy is poor, and that society needs better education on autosomal recessive conditions. This will be challenging, stakeholders warned, given that most people are unaware of their risk:

*“If it’s not something that you see often in your personal surroundings*, *then people will not care about it*. *Everything keeps getting more medical*, *so people will keep wanting to know and be reassured more*. *If you look at it like that*, *more and more people would want to do this*, *but in truth this is not yet our experience*.*”* P4, healthcare provider.

According to some stakeholders, commercial companies that offer direct-to-consumer genetic tests play a crucial role in the growing public awareness concerning DNA and genetics. Some stakeholders expected that genetics, including carrier screening, will become ‘normalized’ to some degree. Subsequently, this will also have an impact on reproductive medicine and reproduction in general, they said. Meanwhile, interviewed members of institutions were hesitant about embedding ECS in national healthcare as a screening program. Reproductive screening can be a sensitive topic, and the government does not want to come across as pushing or ‘sending a message’ (i.e. support for people living with disabilities could be threatened) that screening should be seen as routine. Similar concerns were expressed earlier among members of institutions in the case of NIPT, as well.

### Expectations of Non-invasive prenatal diagnosis (NIPD)

#### Practice: Just a matter of time

At the time of this study, NIPD for monogenic disorders was not yet implemented in Dutch national healthcare (Table 1). Mainly healthcare providers, scientists and some stakeholders working for institutions believed that it is just a matter of time before NIPD will be available in the Netherlands. Some stakeholders were actually surprised that NIPD is not yet available, because it is common for some genetic disorders in other countries such as the United Kingdom. One element is that the NIPD pilot study has made slow progress (partly due to the COVID-pandemic), according to one respondent. Furthermore, the broadening scope of NIPT, which is already implemented in healthcare, may blend diagnostics with screening with a combined NIPT/NIPD in the near future:

*“Then there’s of course the NIPD for monogenic disorders*, *which will be there in the coming years*. *We would initially use this for people who already have an affected child or who are affected themselves*, *so those with a higher risk*. *But if you then extend that*, *then you’ll say*: *yes*, *well*, *if I have an NIPT for chromosomal anomalies*, *why would I not just add a little something for monogenic diseases*?*”* P9, Institution.

A few stakeholders discussed their expectations concerning the use of whole exome sequencing (WES). With this technology, the coding regions (the exons) of all known (disease) genes of a person can be examined at once. Some stakeholders anticipate that this technology will be offered to all pregnant women (via NIPT) in the future. This may facilitate the implementation of NIPD since foetal DNA will also be analysed.

Stakeholders suggested that NIPD could be a safer method to confirm the PGT test result (i.e. PGT pregnancies have a small residual risk of a misdiagnosis of 1 to 2%), given the minimal miscarriage risk with PND. Stakeholders did not believe that NIPD would become a more popular technology than PGT among couples, since PGT is preferred over pregnancy termination. Compared to the currently available prenatal diagnostic technologies, stakeholders listed only advantages of NIPD:

*“The risk of a miscarriage after the results of the chorionic villus sampling or the amniocentesis*, *even if the chance is only 1 in 500*, *people still think it weighs very heavily*, *because it is their decision*. *If it is possible to do without this risk*, *with something just as reliable*, *quick and early in the pregnancy*, *then yes of course you’ll choose that*.*”* P15, healthcare provider.

#### Structure: A few challenges

A few drawbacks and technical challenges were anticipated for NIPD implementation. While stakeholders said that the technology is already very accurate, some challenges were mentioned, such as determining the inherited maternal allele of the foetus, assessing missing genetic information when there is a donor involved, and interpreting the results in case of only a small amount of cell free foetal DNA, in addition to communicating all the information to prospective parents. Moreover, the labour intensity of NIPD was mentioned as a drawback.

#### Culture: No expected need for cultural changes

The implementation of NIPD is not expected to require major changes in the way people think, so stakeholders expressed no need for cultural changes. PND is an accepted technology within society and NIPD would only broaden diagnostic opportunities for women with a known increased risk because the technology is non-invasive.

### Expectations of germline genome editing (GGE)

#### Practice: Far from clinical implementation

Many participants expressed that they expect that GGE is still far from clinical practice. Stakeholders had a wide range of thoughts and perspectives on the developments of GGE. Some believed that GGE will eventually be possible at some point, and will be used when proven safe for human use:

*“If we know how to safely use GGE in humans*, *and I expect that we will in five or ten years*, *then it will happen*. *It already happened […] If we are going to do this*, *it has to be in a safe way*, *and only on genomic diseases*. *But technologically speaking*, *it will definitely be possible*.*”* P1, scientist.

Some stakeholders argued that it is a waste of energy and resources to invest in researching clinical application of GGE in reproductive medicine because there is an already established technology (i.e. PGT) that people will prefer because it is safer and more accepted:

*“I think that people would always prefer the healthy embryo [referring to PGT] over ‘let’s correct the mutation’ [GGE] and then restore the embryo*.*”* P16, institution

#### Structure: Several prerequisites

A stakeholder mentioned that in some countries the field of reproductive technologies is very much privatised and driven by profit rather than social responsibility. In terms of further development and to safeguard human use, stakeholders pointed to current laws and regulations (i.e. Dutch embryo law) that restricts research on human embryos beyond 14 days and bans the creation of human embryos for scientific purposes. It was argued that such laws hinder the development of technologies, like GGE in the reproductive context. Stakeholders had a common ground when it comes to regulation of GGE: good governance and regulation frameworks, nationally and internationally, should be in place to prevent abuse, to safeguard research and possibly guide human application. The costs of the technology were also mentioned: some believed it could be much cheaper than PGT while others believed this decrease in costs will take decades of research. Furthermore, there is a need to decide for what indications GGE would be reimbursed, but those decisions are very complicated, stakeholders said. This is especially difficult because GGE is still far away from clinical application and therefore it is impossible to draft reimbursement frameworks or costs-benefit analysis already. Stakeholders expressed that, in general, reimbursing treatments within reproductive medicine is complicated because the question arises *whom* you would insure, the future child or one of the parents: *“It is actually about insuring a non-existing entity*, *namely an embryo*.*”* P17, institution.

#### Culture: A lot of hesitation

All stakeholders agreed that societal discussions of GGE are crucial, and require engaging all stakeholders involved in the decision-making process to guide further development and potential implementation. It was raised by stakeholders that the hype around GGE could create a *technology push* that we should guard against because some argued that the technology is not yet, and might never be, safe for clinical use. Stakeholders who had a more positive attitude towards GGE suggested that society just needs time to get used to this heavily debated technology, and that people will eventually understand the benefits of it:

*“GGE slippery slope*? *No*, *it’s just further development*. *I mean choosing your car over your bicycle is also a slippery slope*. *What you see is that at a certain point technology becomes more and more normal in your daily life*.*”* P7, industry professional.

Stakeholders who were hesitant to believe that GGE will actually become available for human use brought up several topics such as designer babies and creating ‘superhumans’.

*“I’m not a supporter of this*, *there are too many unanswered questions*. *And aside from that*, *we are moving towards the creation of the Übermensch and designer babies*.*”* P15, healthcare provider.

GGE was often compared with PGT. Some stakeholders believed it could be a ‘PGT+’: for couples in which PGT is not successful, GGE could increase the success (pregnancy) rate. People who had moral objections to discarding embryos and therefore refrain from PGT could opt for GGE as a reasonable alternative. However, one respondent stated that selection will be part of a GGE procedure as well.

Stakeholders who were opposed to GGE believed its introduction would make it hard to draw a line between treatment and enhancement:

*“I am an advocate for retaining the (distinct) medical line between enhancement and healing*. *If you cross that line*, *then I do not think that the added value of the germline editing is big enough for me to say*: *oh yes*, *let’s lift/abolish the ban*! *Because yes*, *I think that with PGT we can really do almost everything*, *with the exception of a few tragic cases*.*”* P20, scientist.

## Discussion

By elucidating and clarifying the perspectives and expectations among Dutch professional stakeholders involved in reproductive and genetic care, this study contributes to an increased understanding of the prerequisites and challenges for responsible introduction of new (future) reproductive technologies (ECS, NIPD, GGE). From a constellation perspective, the expected and desired actions for responsible implementation of innovations in the reproductive healthcare field are described in terms of changes in practice, structure and culture [23].

### Expected implementation of ECS, NIPD and GGE

Stakeholders envisioned different scenarios for the implementation of the three reproductive technologies reviewed here. For ECS, several structural changes may be necessary in order to become established in preconception care. More professionals will have to be trained to provide counselling, and reimbursement of the test will be needed to safeguard equal access, among others. Concerning NIPD, some stakeholders were surprised that this technology is not yet embedded in care as in other countries and all expected it will be as a matter of due course. In the prenatal context, few structural hurdles were identified for NIPD implementation, and availability for couples with a known increased risk seemed feasible on the short term. Expectations and views on GGE varied widely among stakeholders, but all believed that this technology is still far from clinical implementation. For GGE, structural changes were needed in the legal context to shape good governance.

### The Dutch approach: Slow but steady?

The results of this study show that the Dutch cultural background heavily influences the course of new technology implementation. The “Dutch approach” can be characterized by several aspects such as the value of *inclusive dialogues* and the importance of a *learning phase*. Although it is sometimes perceived as unnecessarily viscous, this Dutch approach also has advantages such as building public support and opportunities to learn from small-scale implementation. These aspects have specific advantages and disadvantages that are weighed differently among interviewed stakeholders. The insights gained from this method can also inspire and educate other countries.

Recently, the World Health Organization published recommendations for governance and oversight of human genome editing [30] and one of their recommendations was to facilitate an inclusive global dialogue. The ethical committees of the WHO and the European Commission furthermore recommended establishing a GGE-registry platform, where GGE research knowledge can be shared and used to help create oversight. As Turocy et al [31] suggested, in their review on the progress and considerations of GGE, the way in which mitochondrial replacement therapy was handled in the United Kingdom could serve as a model. In implementing that technology, regulatory and ethical discussions involving stakeholders proved crucial for public acceptance of this therapy. Such a national dialogue on GGE has taken place in the Netherlands in 2019–2020, funded by the government [21]. Soliciting societal input and reaching consensus through public dialogue fits readily within the careful and reasoned Dutch approach. Indeed, the traditional “polder model” was founded on the involvement of all stakeholders to make decisions how to safeguard reclaimed land against flooding [32]. This method of consensus decision-making and cooperation despite differences continues to serve as an example for policy making internationally. Because GGE affects subsequent generations, GGE would benefit from a strong governance model with international oversight and cooperation. “Poldering”, the slow decision-making process where all parties need to be heard, could be perceived justified or, alternatively, as a waste of time or harmful, e.g. when consensus is unfeasible or it unnecessarily delays safe implementation.

Introduction of new technologies in an existing system could benefit from a learning phase, as brought forward by professionals in this study and previously described by van Schendel et al. for NIPT implementation [33]. Especially in the reproductive context, a careful implementation of potentially risky technologies that includes a pilot study and a learning period with suitable follow-up is important to determine adverse effects and establish safety. Unfortunately, history shows that some technologies were implemented prematurely, such as preimplantation genetic screening (PCS): a technology used to screen IVF derived embryos for aneuploidies. Eventually PCS was also offered in clinica to increase the IVF success rate, however this was not properly evaluated with thorough effectiveness studies beforehand, as previously argued [1]. A collective learning phase is considered valuable because participants can gain experience and anticipate structural changes. From the field of transition management and innovation science, we know that emerging innovations often first take place in a ‘niche’. Those niche developments are described by Rotmans et al. as involving individual actors or technologies that can change the current practice of the social-technological landscape [34]. Successful niche developments need scaling up to gradually expand. The two currently available ECS initiatives in the Netherlands can serve as examples of a learning phase and evaluation might provide key insights to consider for potentially scaling up implementation [35,36]. It was expected by professionals in this study that the use of ECS will increase in the future, although currently carrier screening is not widely available and public awareness is low [37]. Lessons learned from global initiatives could be shared, such as ‘Mackenzie’s mission’ in Australia [38]. The Australian federal government funds this pilot study that aims to screen 10,000 couples for 1,200 severe, childhood-onset genetic conditions in a research setting for three years.

### International context: Structural and cultural differences, variable offers

Technologies are embedded in local practice, and are shaped by a national context where culture and structure define the reproductive healthcare system [23]. The core values of the “Dutch approach” shape the implementation of technologies, and this in particular distinguishes the Netherlands from many other countries where healthcare is often, to a larger extent, provided by commercial and private parties. Variability between countries was previously described in studies involving NIPT and ECS [11,18]. An example is the high number of commercial providers in the United States that offer carrier screening [39]. Once high throughput sequencing was available at a reasonable price, commercial companies seized the opportunity to offer large screening panels to the general public [18]. The difference in healthcare systems could also be an explanation for the variability of ECS between countries [40,41]. The Belgian reproductive healthcare system, for example, is differently organised compared to the Netherlands. Belgian women visit a gynaecologist on a regular basis, which simplifies opportunities for preconception counselling and increases awareness of the availability of ECS. Since 2017, the Belgian Superior Health Council recommended to offer ECS for the general population and currently this is available from the clinical genetic centres for all couples at their own cost [42]. Given the difference between healthcare systems in countries it is not possible to simply implement elements a reproductive genetic healthcare system from one country in another country. It is important to understand the contexts of innovation, by unravelling the culture, structure and practice.

### Important policy challenges

The main challenges of current policy seem to be the balance between accessible care for all, while keeping healthcare affordable. The careful consideration of evolving technologies and the egalitarian approach with a strong emphasis on equal access were mentioned in this study as important core values of the “Dutch approach”. Previous evaluation studies of ECS in the Netherlands showed that a large number of participants considered the costs of the test too high and believed that it should be reimbursed by health insurance companies [43,44]. The egalitarian approach favoured by the government is not achieved when out-of-pocket costs hamper equity in access. Whether reimbursement for all could be feasible was discussed by experts at a workshop held at the ESHG in 2015 on ECS [45]. Those experts agreed that equity of access was important, however, they also expressed doubts whether it was feasible because of the high cost of healthcare. The goal of an egalitarian society to avoid unequal access could delay implementation and scaling up innovative discoveries from niches. Another challenge could be in defining the right actors for the implementation process, especially key stakeholders and/or so-called change-agents. Stakeholders often said it was the government’s job to attune roles and responsibilities, and not many participants mentioned their own potential role in the process of responsible implementation. Conversely, the government also expects experts from the field to initiate and orchestrate these matters, which is a discrepancy that hides a potential structural problem.

### Strengths and limitations

This qualitative research enabled rich and in-depth insights into the perspectives of various stakeholders involved in the field of genetic and reproductive health. Insights in what stakeholders view as prerequisites for responsible implementation of technologies in reproductive healthcare in a national context could also be relevant for developments in other countries. A broad range of professional stakeholders were included, which provided an extensive overview of expectations towards the technologies and the changing field. However, completeness of perspectives cannot be assured: general practitioners were approached several times per email but did not respond, and other perspective, e.g. the perspectives of health insurance companies, are missing, which we believe could have contributed valuable insights for the current discussion. All interviewed stakeholders are operating within the ‘Dutch approach’, perhaps a majority has the opinion the system they are supporting is doing things the right way. It would be informative to ask an expert perspective on the ‘Dutch approach’ from professionals in, for example, the United Kingdom, Iceland, or culturally more distinct country for example in Southeast Asia.

## Conclusion

This study presents the perspectives of professional stakeholders from genetic and reproductive healthcare on three emerging technologies: ECS, NIPD and GGE. The general conclusion was that a careful and step-wise implementation of new technologies, referred to as the “Dutch approach”, is desirable for achieving social acceptance and responsible use of innovative discoveries in line with existing practice, structure and culture. NIPD and ECS are expected to become widely available in the near future and in the case of NIPD with relatively few structural changes. The riskier technologies, like GGE, require a collective learning process, legal framework and inclusive dialogues on a national scale. Though the careful process of introducing new technologies can be perceived by some as an unnecessary waste of time, and one must be alert that useful innovations are not unduly hindered by the complexity of the system. Continued attention should be paid to support sustainable processes for the responsible introduction of reproductive and genetic innovations.

## Supporting information

S1 Checklist Consolidated criteria for reporting qualitative studies (COREQ): 32-item checklist. (DOCX)
