# Supplementary material for: Dynamics of reproductive genetic technologies: Perspectives of professional stakeholders
Source: PLoS One. 2022 Jun 21;17(6):e0269719. doi: 10.1371/journal.pone.0269719 (PMC9212161; doi:10.1371/journal.pone.0269719)
Supplement: S1 Checklist — (DOCX) [file pone.0269719.s001.docx]

**Consolidated criteria for reporting qualitative studies (COREQ): 32-item checklist**

Developed from:

Tong A, Sainsbury P, Craig J. Consolidated criteria for reporting qualitative research (COREQ): a 32-item checklist for interviews and focus groups. *International Journal for Quality in Health Care*. 2007. Volume 19, Number 6: pp. 349 – 357

**PROVIDE A RESPONSE FOR ALL ITEMS. ENTER N/A IF NOT APPLICABLE**

| **No. Item** | **Guide questions/description** | **Reported on Page #** |
| --- | --- | --- |
| **Domain 1: Research team and reﬂexivity** |  |  |
| *Personal Characteristics* |  |  |
| 1. Interviewer/facilitator | Which author/s conducted the interview or focus group? | The first author (I.D) conducted all the interviews. |
| 2. Credentials | What were the researcher’s credentials? E.g. PhD, MD | MsC |
| 3. Occupation | What was their occupation at the time of the study? | PhD student |
| 4. Gender | Was the researcher male or female? | Female |
| 5. Experience and training | What experience or training did the researcher have? | Masters education were qualitative research skills were gained and other qualitative studies during PhD trajectory. |
| *Relationship with participants* |  |  |
| 6. Relationship established | Was a relationship established prior to study commencement? | No there was not. |
| 7. Participant knowledge of the interviewer | What did the participants know about the researcher? e.g. personal goals, reasons for doing the research | The participants received an information letter once confirming their interest in participating in the research. |
| 8. Interviewer characteristics | What characteristics were reported about the interviewer/facilitator? e.g. Bias, assumptions, reasons and interests in the research topic | -Researcher Introduced herself in interviews as researcher from Amsterdam UMC/ Human genetics department. |
| **Domain 2: Study design** |  |  |
| *Theoretical framework* |  |  |
| 9. Methodological orientation and Theory | What methodological orientation was stated to underpin the study? e.g. grounded theory, discourse analysis, ethnography, phenomenology, content analysis | Page 5 of manuscript- Two theoretical frameworks were used for the study design and the interpretation of findings. Thematic content analysis was performed |
| *Participant selection* |  |  |
| 10. Sampling | How were participants selected? e.g. purposive, convenience, consecutive, snowball | Page 6- purposive sampling was used |
| 11. Method of approach | How were participants approached? e.g. face-to-face, telephone, mail, email | Page 6- Mail- and email |
| 12. Sample size | How many participants were in the study? | Page 6, a total number of 21 participants. |
| 13. Non-participation | How many people refused to participate or dropped out? Reasons? | Page 6-Seven participants, four did not consider themselves suitable for participation and three did not respond to email invitations. |
| *Setting* |  |  |
| 14. Setting of data collection | Where was the data collected? e.g. home, clinic, workplace | At the workplace of the participants (n=5)and due to the coronavirus some interviews were conducted via video call (n=16). |
| 15. Presence of non-participants | Was anyone else present besides the participants and researchers? | No |
| 16. Description of sample | What are the important characteristics of the sample? e.g. demographic data, date | Is described in the results section |
| *Data collection* |  |  |
| 17. Interview guide | Were questions, prompts, guides provided by the authors? Was it pilot tested? | Page 7 kun je iets van opschrijven? |
| 18. Repeat interviews | Were repeat inter views carried out? If yes, how many? | No, non. |
| 19. Audio/visual recording | Did the research use audio or visual recording to collect the data? | Yes, audio recording |
| 20. Field notes | Were ﬁeld notes made during and/or after the interview or focus group? | Yes, but not written down in methods |
| 21. Duration | What was the duration of the inter views or focus group? | Approximately 45 minutes |
| 22. Data saturation | Was data saturation discussed? | Yes |
| 23. Transcripts returned | Were transcripts returned to participants for comment and/or correction? | No transcripts were not returned. Although, it was clearly explained to participants that we would use their quotes (or at least some) in our results. All participants indicated that they would be interested to receive a copy of the article after publication. |
| **Domain 3: Analysis and ﬁndings** |  |  |
| *Data analysis* |  |  |
| 24. Number of data coders | How many data coders coded the data? | 2 |
| 25. Description of the coding tree | Did authors provide a description of the coding tree? | No |
| 26. Derivation of themes | Were themes identiﬁed in advance or derived from the data? | Both deductive and inductive. |
| 27. Software | What software, if applicable, was used to manage the data? | Altas ti. |
| 28. Participant checking | Did participants provide feedback on the ﬁndings? | No |
| *Reporting* |  |  |
| 29. Quotations presented | Were participant quotations presented to illustrate the themes/ﬁndings? Was each quotation identiﬁed? e.g. participant number | Yes, in the tables. |
| 30. Data and ﬁndings consistent | Was there consistency between the data presented and the ﬁndings? | Yes, similarities were described. |
| 31. Clarity of major themes | Were major themes clearly presented in the ﬁndings? | Yes, the themes were presented in the results section and in associated tables. |
| 32. Clarity of minor themes | Is there a description of diverse cases or discussion of minor themes? | Yes these cases were described. Hier nog voorbeeld geven? |
